# Easy Access to Biomedicine and Knowledge about Medicinal Plants: A Case Study in a Semiarid Region of Brazil

**DOI:** 10.1155/2022/5073625

**Published:** 2022-07-21

**Authors:** Bruno Melo de Sousa, Ulysses Paulino Albuquerque, Elcida de Lima Araújo

**Affiliations:** ^1^Programa de Pós-Graduação Em Botânica, Universidade Federal Rural de Pernambuco, Rua Dom Manuel de Medeiros, S/n-Dois Irmãos, Recife, PE 52171-900, Brazil; ^2^Departamento de Botânica, Universidade Federal de Pernambuco, Av. Prof. Moraes Rego, 1235-Cidade Universitária, Recife, PE 50670-901, Brazil

## Abstract

We aimed to evaluate how proximity to urban areas interferes with the relationship between socioeconomic variables and various aspects of medicinal plant knowledge. The study was conducted in six communities of the Catimbau National Park (PARNA Catimbau) in the state of Pernambuco. Eighty participants were interviewed. The communities were divided into two groups according to their distance from the nearest urban center. Socioeconomic data and information on medicinal plants were collected through semistructured interviews. Subsequently, generalized linear models were generated to verify the influence of the interaction between the variables on medicinal knowledge. We observed that proximity to the urban center influenced the relationship between the level of education and the knowledge of body systems treated by medicinal plants. We concluded that environmental variables can generate a differentiated effect on the influence of socioeconomic factors on one's knowledge of medicinal plants.

## 1. Introduction

Traditional medicine and biomedicine are two branches of medicine with differing characteristics. Traditional medicine is based on popular knowledge (obtained from experiments and experiences of human populations) on the use of medicinal resources and reflects the cultural practices, beliefs, and socioeconomic factors of a given population. In contrast, biomedicine is characterized by clinical and experimental studies that outline scientifically proven ways of treating diseases and involves medical consultations, laboratory tests, and the use of medicines and pharmaceuticals [1, 2].

Traditional medicine is more common in rural areas due to the greater availability of medicinal plants in those regions than in urban environments and its greater economic and physical accessibility (compared to that of biomedical services) for rural communities [3]. Biomedicine, on the other hand, is more likely to be practiced in urban areas [4–6] as accessing medical appointments, hospitals, allopathic medicines, and pharmacies may be easier in those areas than in rural areas [7, 8]. The close proximity of rural areas to urban centers can favor hybridization between these different types of medicine and result in biomedical science interacting with the knowledge of local/traditional medicine [9, 10].

Hybridization is the process by which different types of medicine coexist; it can affect the knowledge and use of traditional medicine through the substitution of a medicinal plant for an allopathic medicine or complement the treatment of diseases [11]. Additionally, socioeconomic factors can influence hybridization [12–14] as well as medicinal knowledge, as demonstrated in the following paragraphs.

Quinlan and Quinlan [15] found that individuals working in the trade had greater knowledge about medicinal plants than those who did not. In addition, when analyzing the combined effects of general occupation and formal education level, they showed that individuals with a commercial occupation and higher formal education level possessed less knowledge about medicinal plants. These results indicate that different variables can modulate medicinal knowledge.

Although it has been shown that an individual's sex and education level may be correlated with their knowledge of local medicine, this varies across geographic regions and income levels. Higher education levels generally provide better-paying jobs [15–17]; this can lead to individuals having greater purchasing power with respect to allopathic medicines (pharmaceuticals) and a closer approach to biomedicine. In rural communities, women are generally responsible for the healthcare of family members, which entails them having greater knowledge about medicinal resources than that possessed by men in their communities [18]. Currently, the impact of access to biomedicine on knowledge of local medicine and how that might vary with an individual's sex and level of education is not understood.

Some studies have shown that the elderly are more likely to prefer traditional medicine over biomedicine [19, 20] and generally have greater knowledge about traditional medicine than young individuals [21–23]. However, the impact of the proximity to biomedicine, if any, on this knowledge is not clear. In other words, do older people with greater access to biomedicine have less knowledge of medicinal plants than people of a similar age with less access to biomedicine?

Similarly, the impact of the number of cohabitants in residences on their medicinal knowledge has not yet been explored in depth. Andriamparany et al. [3] found a positive correlation between the number of residents in a household and their use and knowledge of medicinal plants. However, Alqethami et al. [24] found no such correlation. This divergence in results highlights the need for further studies like these and/or investigations into other potential factors that may interact with the number of cohabitants in a given residence and influence their use and knowledge of medicinal plants.

Different types of relationships can occur between one's socioeconomic characteristics (e.g., gender and occupation) and their knowledge of medicinal plants [18] (see also Medeiros et al. [25] and García et al. [26]). Furthermore, access to biomedicine favors the combined use of biomedicine and traditional medicine [12]. This combination leads us to question whether close proximity to urban centers can modify the predictive force between socioeconomic variables and consequently modify its influence on the knowledge and use of medicinal plants. Assessing this question is of significant importance because it allows for a better understanding of not only the predictive role of socioeconomic variables in the relationship between people and the use of medicinal plants but also in the structuring of local medical systems.

Hence, in this study, we aimed to assess how proximity to biomedicine influences the relationship between socioeconomic variables (age, gender, education, and number of inhabitants per residence/house) and certain aspects of medicinal knowledge (number of known medicinal plants, therapeutic targets/treatments, and body systems treated by medicinal plants).

## 2. Materials and Methods

### 2.1. Study Area

**Figure 1:**
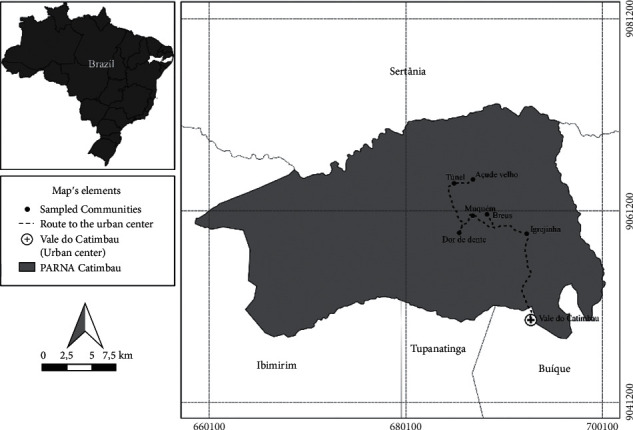
Map of the delineation of PARNA Catimbau in the semiarid region of Brazil, indicating the location of the communities studied and the path taken (dashed line) by people to the urban center of Vale do Catimbau.

The study was conducted in six communities located within Catimbau National Park (PARNA Catimbau): Sítio Igrejinha, Breus, Dor de dente, Muquém, Túnel, and Açude Velho. PARNA Catimbau is located in the state of Pernambuco, between the geographic coordinates 8°29′01.7″S and 37°20′08.3″W, has a total area of 62,294.10 ha, and covers three municipalities: Buíque, Tupanatinga, and Ibimirim (Figure 1). A total of 325 inhabitants, with approximately 200 adults and 125 minors, were distributed from 109 families living in the six selected communities. PARNA Catimbau was established as an environmental protection area in 2002; however, these communities were living there prior to this establishment. Land expropriation processes have not been completed and have generated local socio-environmental conflicts [27, 28].

The region is a semiarid type of climate (type BSh) [29], according to the Köppen classification. The total rainfall varies between municipalities but generally has an annual average of less than 700 mm, and the annual average temperature is approximately 23°C. The vegetation consists of a mosaic of arboreal and shrubby Caatingas. Some of the dominant plant families found at the site are Fabaceae, Euphorbiaceae, Boraginaceae, Cactaceae, Malvaceae, Bromeliaceae, and Asteraceae [28, 30].

### 2.2. Profile of the Populations of PARNA Catimbau

Most of the local population live below the World Bank poverty line ($1.90 US dollars/person/day) and are highly dependent on forest resources. The main occupation in the region is related to agriculture. However, most families' incomes come from the sale of animals, especially that of goats. Several families within the park receive support (such as food, clothing, and medicine) from nongovernmental and governmental organizations, such as the Bolsa Família Program (government aid distributed monthly to people in poverty and extreme poverty). More than half of their homes are built with mud and wood and do not have sewers or toilets. Streets are either paved or unpaved [27, 28].

Families living within PARNA Catimbau are isolated because of the distance from markets and commerce in urban areas. In addition, as they live within environmental protection areas, they are more susceptible to poverty due to land use restrictions [28]. The closest urban center to PARNA Catimbau is Vale do Catimbau, where community members travel 25 min to 1 h to access schools and/or health posts because of the lack of such facilities within PARNA Catimbau. In addition, since 2005, a nongovernmental organization called Amigos do Bem has offered the families of PARNA do Catimbau employment opportunities through plantation of cashew and nut processing as well as housing that is situated closer to schools with basic and complementary education [31].

Taking the urban center of Vale do Catimbau as the closest place that allowed people access to biomedicine, i.e., allopathic medicines and medical consultations, the distance of each community was calculated using Google Maps and GPS. We did not consider the travel time of residents to the urban center as all travel is via a single main unpaved route. Motorized vehicles are the common mode of transport; therefore, a shorter distance reflects a shorter travel time. The proximity of the six communities to Catimbau Valley ranged from 11 to 22.9 km. Among these communities, Igrejinha, which has the largest number of inhabitants, is closest to the Catimbau Valley, being only 11.3 km from this urban center. The other communities are located between 15.6 km and 22.9 km from Vale do Catimbau.

### 2.3. Ethical Legal Aspects

This study was approved by the Ethics and Research Committee of the Federal University of Pernambuco (CAE 40412318.4.0000.5207). Before data collection, all the participants were informed of the study objectives. Those who agreed to participate in the study were asked to sign the free and informed consent term to meet the legal requirements of research involving human beings according to the current legislation of the National Health Council (Resolution No. 510/2016).

### 2.4. Data Collection, Tabulation, and Statistical Analysis

All homes in the six communities were visited to conduct a sample census. Houses without residents or those with residents who insisted on interviewing at another time were excluded from the sample after the third attempt. In each community, structured interviews were initially conducted with all residents aged >18 years, with a total of 102 informants. During the interview, information was on socioeconomic factors such as age, gender, employment, number of people per household, family income, and education was collected. Then, using the free-list technique, each informant was asked to indicate the medicinal plants they knew and what purposes they had used them for.

A preliminary analysis of the collected data was then carried out, and it was found that seven informants did not indicate some of the socioeconomic data, especially those related to income. A possible reason for this is the local conflict that was generated with the creation of the park, which has caused fear in the informants about possible expropriation of their lands. Therefore, we chose not to include the family income variable in the analysis to avoid reducing the sample size. In addition, 15 informants did not list medicinal plants and admitted in this study that they did not know or did not want to divulge this information. We aimed to assess whether the relationship of each variable with local medicinal knowledge is modified when we consider the interaction between them and the distance from the urban center, so we chose to include only individuals who demonstrated knowledge about medicinal plants in the sample. Consequently, two criteria were adopted to define the final sample of informants to be considered in our study: (a) having made available all socioeconomic data and (b) having answered the free list on medicinal plants. Based on these criteria, the responses of only 80 participants were assessed.

We considered the central distance of displacement in each distance group, along the central route (only the local path), to the nearest urban center with access to biomedicine, as a measure of access to biomedicine. We assumed that the community closest to the Catimbau Valley overcomes the distance barrier more easily and has easier access to biomedicine than communities farther away from the Catimbau Valley. The most distant group (15.6 to 22.9 km) from Vale do Catimbau included informants (*n* = 25) from the communities Açude Velho, Breus, Dor de dente, Muquém, and Tunnel. Meanwhile, the closest group (11.3 km) comprised informants (*n* = 55) from the Igrejinha community.

The age of an individual and number of people per household were not categorized. Interviewees' formal education level was calculated using the number of academic years taken from the first grade of elementary school. For example, an informant who studied up to the fourth grade of elementary school would have had four years of formal education. There were no informants with a higher education or graduate degree (complete or incomplete). Sex and distance to the urban center were tabulated as discrete numerical variables as follows: 1 for females or informants from the close group and 2 for male informants or informants from the distant group.

Although biomedicine considers the symptoms of diseases mentioned by the interviewees as mentioned of diseases and not necessarily mentioned therapeutic complaints as a disease, in this study, the diseases and/or symptoms treated medicinally were considered as medicinal targets, representing the informants' perception of diseases treated with medicinal plants, drawing from [32]. Therapeutic targets were classified into body systems based on the International Statistical Classification of Diseases and Related Health Problems 10th Revision [33]. Therapeutic targets of religious nature were categorized as spiritual diseases. After classification, the number of body systems treated with the medicinal plants of each informant was counted. The measures used as response variables to assess the influence of social variables on knowledge of medicinal plants among the distance groups were the number of known medicinal plants (treated as ethnospecies/vernacular name), the number of therapeutic targets cited by the informant, and the number of body systems treated by medicinal plants.

To test the hypothesis that ease of access to the urban center can modify the explanatory power of the interactions of social variables on the knowledge of medicinal plants, generalized linear models (GLMs) were performed. For each dependent variable, models were created with and without interactions between the independent variables in addition to a null model. For each model, the Akaike information criterion corrected (AICc) for a small sample [34] and ΔAICc were calculated. Models with ΔAICc >2 were selected [35]. Reduction in the selected models was performed using the likelihood method. The variables, number of ethnospecies, and number of therapeutic targets were transformed into square roots and a Gaussian error distribution was used to create the models. For a variable number of body systems, a Poisson distribution was used to create the models. The independent variables in the GLMs were age, sex, number of inhabitants per household, and education level. We chose not to include informants' occupation as an independent variable in the GLMs because of the lack of information from some informants and the consequent reduction of sample size. All statistical tests were performed using the R software [36].

## 3. Results

Models with the interaction of variables, such as the number of therapeutic targets and number of ethnospecies, did not prove to be plausible models but were likely to occur (ΔAICc >2) (Table 1).

Age was the only social variable that influenced the informants' knowledge of ethnospecies and therapeutic targets. Old adults showed greater knowledge of ethnospecies and therapeutic targets than that by young adults (Table 2).

Distance from the urban center did not influence the knowledge of the number of ethnospecies and therapeutic targets, i.e., people from communities both near and far from the urban center had similar knowledge about ethnospecies and therapeutic targets. However, the distance from the urban center, when interacting with education level, influenced the knowledge of body systems treated by medicinal plants among the informants. In other words, people with higher education and from communities far from the urban center knew a greater number of body systems treated by plants compared with the people with higher education from communities near the urban center (Table 2). However, when analyzing the influence of education separately, we found that people with higher education cited fewer body systems treated with medicinal plants. Distance from the urban center alone did not have a considerable influence on the number of body systems treated with medicinal plants.

## 4. Discussion

By analyzing different measures of knowledge, we were able to show that elderly people possessed greater knowledge of ethnospecies and therapeutic targets in Catimbau. This can be explained by the fact that older people have had greater opportunity to try different medicinal species for the same therapeutic purpose and/or experience a greater diversity of diseases in the course of life compared to younger people. In addition, older people may be more likely to try different medicinal plants than that by younger people, as indicated by previous studies [37–39]; this may occur locally and generate greater medicinal knowledge.

We expected that a greater number of cohabitants would diversify the experience of family members in the use of medicinal plants for the treatment of diseases, which is consistent with the findings of Alqethami et al. [24]. Our findings suggest that other factors may modulate this relationship.

Gender is recorded as a variable that can directly or indirectly affect knowledge about medicinal plants as it is associated with other factors, such as the type of work performed by men and women [12]. Studies have reported that in communities where women play the role of caretakers and are responsible for collecting medicinal resources, they have a greater plant knowledge [18, 40–42]. However, if a man is involved in agricultural activities and is responsible for collecting medicinal resources from forests, his knowledge of medicinal plants may be more expansive than that of the women [27]. Sometimes, men perform the role of healers in some indigenous Brazilian communities, which means that their knowledge of medicinal plants is more extensive than that of the women [18]. However, some studies have shown no relationship between sex and knowledge of medicinal plants [37, 43, 44], as was found in our study for the communities studied in Catimbau.

In general, the advancement of schooling enables jobs with better pay [15] (see also Júnior et al. [16]), which can increase individuals' purchasing power and favors the acquisition of pharmaceutical drugs. Indirectly, the increase in schooling may have a negative effect on the knowledge and use of medicinal plants, as reported in several studies [15] (see also Kidane et al. [44]; Teka et al. [45]; Voeks and Leony [46]). A greater purchasing power of pharmaceutical drugs reduces people's dependence on natural medicinal resources [47]. However, we did not find a relationship between the number of known species and the level of education, which is consistent with findings of other studies [48, 49]; we recorded a decrease in the knowledge of the number of body systems treated by medicinal plants with the advancement of schooling. This decrease in the knowledge of body systems treated with medicinal plants may be related to the health of the individual, as a previous study suggests that healthier individuals tend to have a higher level of education [50], but this needs to be tested with the methods used in our study.

In addition, accessing schools requires the residents of Catimbau to relocate to nearby urban centers (Vale do Catimbau and/or Buíque), which may provide greater contact with biomedicine. Consequently, the inclusion of biomedicine in the treatment of diseases can, over time, interfere with knowledge about medicinal plants, as people may prefer the use of biomedicine and retain less knowledge about medicinal plants, as recorded in the study by Alqethami et al. [24]. People with higher education are likely to have greater contact with biomedicine, directly influencing the choice between the use of medicinal plants and biomedicine. Moreover, people with higher education levels are more familiarized with and/or educated about the treatment of biomedicine for new therapeutic targets compared with the people with less education, such as those in the Catimbau region.

Many rural communities lack basic health services and inhabitants are forced to travel to urban centers to access them. This physical isolation can provide residents with greater knowledge of medicinal plants by increasing their dependence on them [9] (see also García et al. [51]). Moreover, contact with biomedicine may not interfere with knowledge of medicinal plants [7, 11] (see also Hoyler et al. [52]; Stiefel et al. [53]; Vandebroek and Balick [54]). In Catimbau, the distance of isolation did not influence the three aspects of medicinal knowledge analyzed in this study; however, the interaction of distance from the urban center with schooling proved to impact an individual's medicinal knowledge.

The interaction between schooling and distance to urban centers positively influenced knowledge about the number of body systems treated by medicinal plants, i.e., informants from distant communities with higher education knew a greater number of body systems treated by medicinal plants than informants with higher levels of school education from nearby communities. An increase in schooling likely favors better knowledge of the characteristics and symptoms of some diseases, which can improve the targeting and choice of plants to treat a disease in specific parts of the human body. However, this knowledge needs to be incorporated into practice so that it becomes assimilated by the community, which may not happen in communities close to the urban center due to greater ease of access to biomedicine. In this study, informants from more distant communities did not mention any pharmaceutical drug for the treatment of a particular therapeutic target as an alternative to replace a particular medicinal plant, which differs from what was observed in the case of informants from communities close to urban centers. In addition, informants from distant communities commented that they had used medicinal plants for the treatment of a specific therapeutic target more recently than had informants from communities in closer proximity to the urban center.

The findings of our study revealed that distance from the urban center can interact with other socioeconomic variables and generate a differentiated effect on the knowledge of medicinal plants, which makes the medicinal knowledge of each community complex. This indicates the importance of considering the set of variables that characterize the social and ecological context in which populations are situated to enhance the accuracy of generating hypotheses about a given community's medicinal knowledge and its implications for understanding the structure and resilience of medical systems. The interaction between individuals can influence knowledge about medicinal plants, and the communities we studied are consisted of related individuals. However, we did not evaluate the interaction between communities in our study. Therefore, we suggest that in future studies, similar approaches should be considered in the analysis.

## Figures and Tables

**Table 1:** Selection of appropriate models with an information-theoretical approach.

Models	ΔAICc	d*f*
*Number of body systems treated by plants*		
Sex + age + education + residents + distance	2.8	6
Sex *∗* distance + age *∗* distance + education *∗* distance + residents *∗* distance	4.8	10
Education + education : distance	1.5	3
1	8.3	1
Age	0.0	2

*Number of ethnospecies*		
Sex + age + educated + residents + distance	4.3	7
Sex *∗* distance + age *∗* distance + education *∗* distance + inhabitants *∗* distance	8.3	11
1	15.2	2
Age	0.0	3

*Number of therapeutic targets*		
Sex + age + educated + inhabitants + distance	3.8	7
Sex *∗* distance + age *∗* distance + education *∗* distance + residents *∗* distance	8.4	11
1	15.0	2
Age	0.0	3

AICc is the small sample-corrected difference in the Akaike information criterion (AIC) between each model and the smallest AIC value. d*f* is the degree of freedom; ^*∗*^ = interaction between variables and influence of each isolated variable; : = interaction between only two variables; 1 = comparison with the intercept.

**Table 2:** Generalized linear model (GLM) of socioeconomic variables in relation to different measures of knowledge about medicinal plants in the Catimbau Valley.

	Estimation	Std. error	*T*-value	Pr (>|*t*|)	AIC
*Ethnospecies*					
Intercept	2.940814	0.102889	28,580	<2e − 16^*∗∗∗*^	217.73
Age	0.026132	0.006002	4,354	4.02e − 05^*∗∗*^	

*Therapeutic targets*					
Intercept	2.636760	2.636760	28.803	<2e − 16^*∗*^	199.02
Age	0.023060	0.005340	4.319	4.57e − 05^*∗∗*^	

*Body systems*					
*The model with interaction*					
Intercept	1.62541	0.06556	24.794	<2e − 16^*∗∗∗*^	342.72
Schooling	−0.06210	0.02042	−3.042	0.00235^*∗∗*^	
Schooling: distant group^1^	0.06801	0.02557	2.660	0.00781^*∗∗*^	

^1^ = difference from the close group, *p* < 0.01, *p* < 0.001.

## Data Availability

The datasets generated during and/or analyzed during the current study are available from the corresponding author on reasonable request.

## References

[1] Assis J., Conceição M., Licença I. (2018). Medicina tradicional no Brasil e em Moçambique: definições, apropriações e debates em saúde pública. *O Público e o Privado*.

[2] Queiroz M. D. S. (1986). O paradigma mecanicista da medicina ocidental moderna: uma perspectiva antropológica. *Revista de Saúde Pública*.

[3] Andriamparany J. N., Brinkmann K., Jeannoda V., Buerkert A. (2014). Effects of socio-economic household characteristics on traditional knowledge and usage of wild yams and medicinal plants in the mahafaly region of south-western Madagascar. *Journal of Ethnobiology and Ethnomedicine*.

[4] Krout J. A., Rathbone-McCuan E., Shreffler J. M. (2001). Access and issues of equity. *The Journal of Rural Health*.

[5] Nelson J. A., Stover Gingerich B. (2010). Rural health: access to care and services. *Home Health Care Management & Practice*.

[6] Zeng D., You W., Mills B., Alwang J., Royster M., Anson-Dwamena R. (2015). A closer look at the rural-urban health disparities: insights from four major diseases in the commonwealth of Virginia. *Social Science & Medicine*.

[7] Peltzer K., Pengpid S., Puckpinyo A., Yi S., Vu Anh L. (2016). The utilization of traditional, complementary and alternative medicine for non-communicable diseases and mental disorders in health care patients in Cambodia, Thailand and Vietnam. *BMC Complementary and Alternative Medicine*.

[8] Rahayu Y. Y. S., Araki T., Rosleine D. (2020). Factors affecting the use of herbal medicines in the universal health coverage system in Indonesia. *Journal of Ethnopharmacology*.

[9] Vandebroek I., Calewaert J.-B., De jonckheere S. (2004). Use of medicinal plants and pharmaceuticals by indigenous communities in the Bolivian Andes and Amazon. *Bulletin of the World Health Organization*.

[10] Zank S., Hanazaki N. (2017). The coexistence of traditional medicine and biomedicine: a study with local health experts in two Brazilian regions. *PLoS One*.

[11] Ladio A. H., Albuquerque U. P. (2014). The concept of hybridization and its contribution to urban ethnobiology. *Ethnobiology Conservation*.

[12] Nascimento A. L. B., Medeiros P. M., Albuquerque U. P. (2018). Factors in hybridization of local medical systems: simultaneous use of medicinal plants and modern medicine in northeast Brazil. *PLoS One*.

[13] Giovannini P., Reyes-García V., Waldstein A., Heinrich M. (2011). Do pharmaceuticals displace local knowledge and use of medicinal plants? estimates from a cross-sectional study in a rural indigenous community, Mexico. *Social Science & Medicine*.

[14] Odonne G., Musset L., Cropet C. (2021). When local phytotherapies meet biomedicine. Cross-sectional study of knowledge and intercultural practices against malaria in eastern French Guiana. *Journal of Ethnopharmacology*.

[15] Quinlan M. B., Quinlan R. J. (2007). Modernization and medicinal plant knowledge in a caribbean horticultural village. *Medical Anthropology Quarterly*.

[16] Júnior W. S. F., Santoro F. R., Vandebroek I., Albuquerque U. P. (2016). Urbanization, modernization, and nature knowledge. *Introduction to Ethnobiology*.

[17] Srithi K., Balslev H., Wangpakapattanawong P., Srisanga P., Trisonthi C. (2009). Medicinal plant knowledge and its erosion among the mien (yao) in northern Thailand. *Journal of Ethnopharmacology*.

[18] Torres-avilez W., De Medeiros P. M., Albuquerque U. P. (2016). Effect of gender on the knowledge of medicinal plants: systematic review and meta-analysis. *Evidence-Based Complement. Alternative Medicine*.

[19] Nega S. S., Bekele H. M., Meles G. G., Nordeng H. (2019). Medicinal plants and concomitant use with pharmaceutical drugs among pregnant women. *Journal of Alternative & Complementary Medicine*.

[20] Sato A. (2012). Does socio-economic status explain use of modern and traditional health care services?. *Social Science & Medicine*.

[21] de Almeida C. D. F. C. B. R., Ramos M. A., de Amorim E. L. C., de Albuquerque U. P. (2010). A comparison of knowledge about medicinal plants for three rural communities in the semi-arid region of northeast of Brazil. *Journal of Ethnopharmacology*.

[22] Dapar M. L. G., Meve U., Liede-Schumann S., Alejandro G. J. D. (2020). Ethnomedicinal plants used for the treatment of cuts and wounds by the agusan manobo of sibagat, agusan del sur, Philippines. *Ethnobotany Research and Applications*.

[23] Godoy R., Reyes-García V., Broesch J. (2009). Long-term (secular) change of ethnobotanical knowledge ofuseful plants: separating cohort and age effects. *Journal of Anthropological Research*.

[24] Alqethami A., Aldhebiani A. Y. A. Y., Teixidor-Toneu I. (2020). Medicinal plants used in Jeddah, Saudi Arabia: a gender perspective. *Journal of Ethnopharmacology*.

[25] Medeiros P. M., Campos J. L. A., Albuquerque U. P. (2016). Ethnicity, income, and education. *Introduction to Ethnobiology*.

[26] García V. R., Kightley E., Ruiz-Mallén I. (2010). Schooling and local environmental knowledge: do they complement or substitute each other?. *International Journal of Educational Development*.

[27] Gonçalves P. H. S., da Cunha Melo C. V. S., de Assis Andrade C. (2021). Livelihood strategies and use of forest resources in a protected area in the Brazilian semiarid. *Environment, Development and Sustainability*.

[28] Specht M. J., Santos B. A., Marshall N. (2018). Socioeconomic differences among resident, users and neighbour populations of a protected area in the Brazilian dry forest. *Journal of Environmental Management*.

[29] Alvares C. A., Stape J. L., Sentelhas P. C., De Moraes Gonçalves J. L., Sparovek G. (2013). Köppen’s climate classification map for Brazil. *Meteorologische Zeitschrift*.

[30] Rito K. F., Arroyo-Rodríguez V., Queiroz R. T., Leal I. R., Tabarelli M. (2017). Precipitation mediates the effect of human disturbance on the Brazilian Caatinga vegetation. *Journal of Ecology*.

[31] Freire N. C. F., Silva J. B., Moura D. C. (2015). Mapeamento e análise espectro-temporal das unidades de conservação de proteção Integral da administração federal no bioma Caatinga-parque nacional do catimbau. *Recife: Fundação Joaquim Nabuco*.

[32] Ferreira-Júnior W. S., da Silva T. G., Alencar Menezes I. R., Albuquerque U. P. (2016). The role of local disease perception in the selection of medicinal plants: a study of the structure of local medical systems. *Journal of Ethnopharmacology*.

[33] World Health Organization (2019). *ICD-11 International Classification of Diseases for Mortality and Morbidity Statistics*.

[34] Akaike H. (1973). Maximum likelihood identification of Gaussian autoregressive moving average models. *Biometrika*.

[35] Burnham K. P., Anderson D. R. (2004). Multimodel inference: understanding AIC and BIC in model selection. *Sociological Methods & Research*.

[36] RC Team (2019). *R. A. Language and Environment for Statistical Computing*.

[37] Hajiali A., Robescu L. D. (2019). *Assessment of Biological Removal of Fe as a Heavy Metal in Wastewater Treatment: Comparison of Active Sludge and Aquatic Fern Usage*.

[38] da Silva N. F., Hanazaki N., Albuquerque U. P., Almeida Campos J. L., Feitosa I. S., Araujo EdL. (2019). Local knowledge and conservation priorities of medicinal plants near a protected area in Brazil. *Evidence-Based Complementary and Alternative Medicine*.

[39] Wiryono W., Wanandi Y., Ilahi A. K., Deselina D., Senoaji G., Siswahyono S. (2019). The local knowledge of the plant names and uses by semende tribe people in Kaur district, Bengkulu province, Indonesia. *Biodiversitas Journal of Biological Diversity*.

[40] Bouasla A., Bouasla I. (2017). Ethnobotanical survey of medicinal plants in northeastern of Algeria. *Phytomedicine*.

[41] Corroto F., Gamarra Torres O. A., Macía M. J. (2018). Different patterns in medicinal plant use along an elevational gradient in northern Peruvian Andes. *Journal of Ethnopharmacology*.

[42] Malinga G. M., Baana K., Rutaro K. (2020). An ethnobotanical study of plants used for the treatment of malaria in budondo sub-county, eastern Uganda. *Ethnobotany Research and Applications*.

[43] Drané E., Rippeault M. F., Ravin J. S., François-Haugrin O. (2018). Ethnobotanical study in Martinique of the species behind the local plant name bwa kaka. *Ethnobiology Letters*.

[44] Kidane L., Gebremedhin G., Beyene T. (2018). Ethnobotanical study of medicinal plants in Ganta Afeshum district, eastern zone of Tigray, northern Ethiopia. *Journal of Ethnobiology and Ethnomedicine*.

[45] Teka A., Asfaw Z., Demissew S., Van Damme P. (2020). Traditional medicinal plant use of indigenous communities in Gurage zone, Ethiopia. *Ethnobotany Research and Applications*.

[46] Voeks R. A., Leony A. (2004). Forgetting the forest: assessing medicinal plant erosion in eastern Brazil. *Economic Botany*.

[47] Vedeld P., Jumane A., Wapalila G., Songorwa A. (2012). Protected areas, poverty and conflicts. *Forest Policy and Economics*.

[48] Ayantunde A. A., Briejer M., Hiernaux P., Udo H. M. J., Tabo R. (2008). Botanical knowledge and its differentiation by age, gender and ethnicity in southwestern Niger. *Human Ecology*.

[49] Gyasi R. M., Mensah C. M., Siaw L. P. (2015). Predictors of traditional medicines utilisation in the Ghanaian health care practice: interrogating the ashanti situation. *Journal of Community Health*.

[50] Lynch J. L., von Hippel P. T. (2016). An education gradient in health, a health gradient in education, or a confounded gradient in both?. *Social Science & Medicine*.

[51] García V. R., Guèze M., Luz A. C. (2014). Evidence of traditional knowledge loss among a contemporary indigenous society. *Evolution and Human Behavior*.

[52] Hoyler E., Martinez R., Mehta K., Nisonoff H., Boyd D. (2018). Beyond medical pluralism: characterising health-care delivery of biomedicine and traditional medicine in rural Guatemala. *Global Public Health*.

[53] Stiefel S.-L. M., Vandebroek I., Rist S. (2012). Can Andean medicine coexist with biomedical healthcare ? a comparison of two rural communities in Peru and Bolivia can Andean medicine coexist with biomedical healthcare ? a comparison of two rural communities in Peru and Bolivia. *Journal of Ethnobiology and Ethnomedicine*.

