# Corrigendum to “Easy Access to Biomedicine and Knowledge about Medicinal Plants: A Case Study in a Semiarid Region of Brazil”

**DOI:** 10.1155/ecam/9893532

**Published:** 2025-08-30

B. M. Sousa, U. P. Albuquerque, and E. L. Araújo, “Easy Access to Biomedicine and Knowledge about Medicinal Plants: A Case Study in a Semiarid Region of Brazil,” *Evidence-Based Complementary and Alternative Medicine* 2020 (2022): 5073625, https://doi.org/10.1155/2022/5073625.

In the article, there are errors in the legend of Table 2. The correct legend is shown below:

Table 2: Generalized linear model (GLM) of socioeconomic variables in relation to different measures of knowledge about medicinal plants in the Catimbau Valley. ^1^ = difference from the close group, ⁣^∗^=*p* < 0.05, ⁣^∗∗^=*p* > 0.01, ⁣^∗∗∗^=*p* > 0.001.

Additionally, reference 54 has been mistakenly omitted. The correct reference 54 [1] is shown below:

I. Vandebroek, and M. J. Balick, “Globalization and Loss of Plant Knowledge: Challenging the Paradigm,” *PLoS One* 7, no. 5 (2012): e37643, https://doi.org/10.1371/journal.pone.0037643.

We apologize for these errors.
